# Dual inhibition of BET and HAT/p300 suppresses colorectal cancer via DR5- and p53/PUMA-mediated cell death

**DOI:** 10.3389/fonc.2022.1018775

**Published:** 2022-10-12

**Authors:** Chaoyuan Kuang, Jingshan Tong, Kaylee Ermine, Manbo Cai, Fujun Dai, Suisui Hao, Francis Giles, Yi Huang, Jian Yu, Lin Zhang

**Affiliations:** ^1^ Hillman Cancer Center, University of Pittsburgh, Pittsburgh, PA, United States; ^2^ Division of Hematology/Oncology, Department of Medicine, University of Pittsburgh, Pittsburgh, PA, United States; ^3^ Department of Pharmacology and Chemical Biology, University of Pittsburgh, Pittsburgh, PA, United States; ^4^ Developmental Therapeutics Consortium, Chicago, IL, United States; ^5^ Department of Pathology, University of Pittsburgh, Pittsburgh, PA, United States

**Keywords:** colorectal cancer, drug development, epigenetics, BET inhibitor, apoptosis

## Abstract

**Background:**

Colorectal cancer (CRC) frequently has a dysregulated epigenome causing aberrant up-regulation of oncogenes such as c-MYC. Bromodomain and extra-terminal domain (BET) proteins and histone acetyltransferases (HAT) are epigenetic regulatory proteins that create and maintain epigenetic states supporting oncogenesis. BET inhibitors and HAT inhibitors are currently being investigated as cancer therapeutics due to their ability to suppress cancer-promoting epigenetic modifiers. Due to the extensive molecular crosstalk between BET proteins and HAT proteins, we hypothesized that dual inhibition of BET and HAT could more potently inhibit CRC cells than inhibition of each individual protein.

**Methods:**

We investigated the activity and mechanisms of a dual BET and HAT inhibitor, NEO2734, in CRC cell lines and mouse xenografts. MTS, flow cytometry, and microscopy were used to assess cell viability. qPCR, Western blotting, and immunofluorescent staining were used to assess mechanisms of action.

**Results:**

We found that NEO2734 more potently suppresses CRC cell growth than first generation BET inhibitors, regardless of the status of common CRC driver mutations. We previously showed that BET inhibitors upregulate DR5 to induce extrinsic apoptosis. In the current study, we show that NEO2734 treatment induces CRC cell apoptosis *via* both the intrinsic and extrinsic apoptosis pathways. NEO2734 increases p53 expression and subsequently increased expression of the p53-upregulated mediator of apoptosis (PUMA), which is a critical mechanism for activating intrinsic apoptosis. We demonstrate that inhibition of either the intrinsic or extrinsic branches of apoptosis partially rescues CRC cells from NEO2734 treatment, while the dual inhibition of both branches of apoptosis more strongly rescues CRC cells from NEO2734 treatment. Finally, we show that NEO2734 monotherapy is able to suppress tumor growth in CRC xenografts by inducing apoptosis.

**Conclusions:**

Our study demonstrates NEO2734 potently suppresses CRC cells *in vitro* and *in vivo* by simultaneously upregulating PUMA and DR5 to induce cell death. Further studies of NEO2734 for treating CRC are warranted.

## Background

Colorectal cancer (CRC) is a major health burden and represents the second leading cause of cancer related mortality in the United States (1). The primary cause of death of CRC patients is complications related to metastatic disease. Metastatic colorectal cancer (mCRC) is usually managed with systemic therapy such as chemotherapy, immunotherapy, and targeted therapy (2). A minority of mCRC are microsatellite instability-high (MSI-H) and are sensitive to immunotherapy (3, 4). However, the vast majority of mCRC are microsatellite stable (MSS) and require chemotherapy. Once patients have progressed on 2 lines of fluoropyrimidine-based chemotherapy, there are few meaningful treatment options remaining. Regorafenib and TAS-102 are both approved for chemotherapy-refractory mCRC, however both of these agents have demonstrated modest improvements in survival when compared to best supportive care (5, 6). Thus, novel treatments for mCRC are urgently needed.

CRC genomes frequently undergo significant epigenetic remodeling compared to non-malignant genomes, with the overall changes called the cancer epigenome (7). These changes usually include alterations in DNA methylation status, post-translational modifications of histone tails, and dysregulated nucleosome positions. The cancer epigenome contains many driver modifications that contribute to oncogenesis, often by silencing tumor suppressors or upregulating proto-oncogenes. c-MYC is frequently described as a target of aberrant transcriptional upregulation due to epigenetic changes in cancer (8, 9). Previous studies have suggested that c-MYC, as well as other oncogenes, can be overexpressed in cancer due to the activity of histone modifying proteins such as histone acetyltransferases (HAT), or histone readers such as bromodomain and extra-terminal domain (BET) proteins (10). HATs add acetyl groups to the lysine tails of histones, which creates a more transcriptionally active gene region (11). BET proteins recognize activated histone tail markers such as lysine acetylation, and recruit additional transcription factors to cause overexpression of nearby genes (12). BET and HAT inhibitors are promising new classes of drugs that are designed to target the cancer epigenome by inhibiting these transcriptionally active gene regions and suppress their oncogenic targets (13).

BET inhibitors have demonstrated preclinical activity against several types of cancer, including leukemia (14), lymphoma (15), breast cancer (16), lung cancer (17), and prostate cancer (18, 19). We have previously shown that BET inhibitors are active against CRC and can potentiate the therapeutic effects of 5-FU (20). Our prior study demonstrated that treatment of CRC cells with BET inhibitors induced apoptosis through upregulation of the death receptor pathway. However, existing studies have largely focused on the first generation of BET inhibitors, which have demonstrated high rates of dose-limiting thrombocytopenia in early phase clinical trials (21). One approach to improve the therapeutic index of BET inhibitors is to combine BET inhibitors with other epigenetic agents that act in a molecularly complementary fashion, such as HAT inhibitors. NEO2734 is a dual BET/HAT inhibitor that has demonstrated activity against leukemia, lymphoma, prostate cancer, and NUT midline carcinoma (22–26). We sought to determine the activity and mechanism of action of NEO2734 in CRC.

## Materials and methods

### Cell culture and treatments

Human colorectal cancer cell lines, including HCT116, HT29, RKO, DLD-1, SW403, SW1463, DiFi, and NCI-H508, were purchased from the ATCC and cultured in McCoy's 5A modified media. SNU-407 was purchased from AddexBio and cultured in RPMI1640 (Invitrogen). NCM356D non-transformed colonic epithelial cell line was purchased from INCELL and cultured in M3 media (INCELL). *DR5*-knockout (KO), PUMA-KO, and p53-KO HCT116 cells were described previously (20, 27). Cells were authenticated by genotyping and analysis of protein expression by Western blotting, and routinely checked for *Mycoplasma* contamination by PCR. All cell lines were maintained at 37°C and 5% CO_2_ atmosphere. Cell culture media were supplemented with 10% defined FBS, 100 U/mL penicillin, and 100 μg/mL streptomycin. For drug treatment, cells were plated in 12-well plates at 20%–30% density 24 hours before treatment. DMSO stocks of NEO2734, CPI-637, I-BET151, OTX015, 5-FU, oxaliplatin, and SN-38 were prepared and diluted in cell culture media before adding to cells. All drugs were purchased from commercial sources, except for NEO2734 and CPI-637 which were provided by NEOMED Therapeutics 1, Inc.

### Cellular thermal shift assay (CETSA)

Binding of NEO2734 to endogenous BRD4 and p300/CBP was analyzed by CETSA based on a published protocol (28). Briefly, cells were treated with NEO2734 or control 0.1% DMSO for 1 hour in T75 flasks. After treatment, cells were harvested, washed once with 1× PBS, resuspend in 750 μL HBSS, and lysed by 4 cycles of freezing (dry ice/ethanol for 5 min) and thawing (37°C for 5 min). Samples were then distributed equally into 0.2-mL PCR tubes and heated for 3 min on a thermal cycler set to a gradient, followed by centrifugation at 13,200× rpm for 15 min and analysis of supernatants by western blotting.

### Transfection and small interfering RNA knockdown

Cells were transfected using Lipofectamine 2000 (Invitrogen) according to the manufacturer’s instructions. Small interfering RNA (siRNA) transfection was performed 24 hours before drug treatment using 200 pmol of control scrambled or *DR5* (5′-AAGACCCUUGUGCUCGUUGUCdTdT-3′) (Dharmacon), siRNA.

### Western blotting

Western blotting was performed as previously described (28) using antibodies listed in **Supplementary Table S1**.

### Real-time RT-PCR

Total RNA was isolated from cells using the Mini RNA Isolation II Kit (Zymo Research) according to the manufacturer’s protocol. One microgram of total RNA was used to generate cDNA using the SuperScript II reverse transcriptase (Invitrogen). PCR was performed with the previously described cycle conditions (20) with primers listed in **Supplementary Table S2**.

### Cell viability assay

Cells seeded in 96-well plates at a density of 5 thousand cells/well were treated with different drugs for 72 hours. Cell viability was evaluated by 3-(4,5-dimethylthiazol-2-yl)-5-(3-carboxymethoxyphenyl)-2-(4-sulfophenyl)-2H-tetrazolium (MTS) assay (Promega) according to the manufacturer’s instructions. Absorption (490nm) was measured using a Wallac Victor 1420 Multilabel Counter (Perkin Elmer). Each assay was conducted in triplicate and repeated three times.

### Luciferase reporter assay

pBV-based p53 luciferase reporter plasmid constructs were described previously (29). To measure reporter activities, cells were transfected with indicated plasmids along with the transfection control β-galactosidase reporter, pCMVβ (Promega). Cell lysates were collected and luciferase activities were measured and normalized as described previously (20). All reporter experiments were performed in triplicate and repeated three times.

### Chromatin immunoprecipitation

Chromatin immunoprecipitation (ChIP) was performed using the Chromatin Immunoprecipitation Assay Kit (EMD Millipore, product#) according to the manufacturer’s instructions. The precipitates were analyzed by PCR using the primers listed in **Supplementary Table S2**.

### Apoptosis assays

Apoptosis was measured by counting cells with condensed and fragmented nuclei after nuclear staining with Hoechst 33258 (Invitrogen) as described previously (30). At least 300 cells were analyzed for each group. Apoptosis was also analyzed by flow cytometry of cells stained with Annexin V-APC/propidium iodide (BD). Viable cells in 12-well plates after drug treatment for 36 hours were visualized by staining with crystal violet in 10% neutral buffered formalin (Sigma). Colony formation was assayed by plating equal numbers of 24-hour drug-treated cells in 12-well plates at appropriate dilutions with no drug, followed by visualization of colonies by crystal violet staining after 14 days, and counting of colony numbers using ImageJ, version 1.52a.

### Cell cycle profile

HCT116 cell were cultured in 12-wells and once they reached 30-40% density, they were treated with NEO2734 10 µM or with media only control. After drug treatment, all adherent cells were detached with trypsin-EDTA, pooled with the non-adherent cells, washed once with HBSS, and fixed with ice cold 70% ethanol for 2 hours. Fixed cells were pelleted and resuspended in a staining solution (propidium iodide 5µg/mL, PBS, and RNAse A), and analyzed by flow cytometry immediately (BD Accuri C6, FlowJo v10.8). Using FlowJo software, cellular debris and doublets were excluded based on forward scatter and side scatter gating. FlowJo cell cycle analysis tool based on the Watson Model was applied to the samples. Curves were optimized and fitted to root-mean-squared differentials of 1.70 (untreated) and 2.33 (NEO2734 treated) samples (see supplemental raw data). The calculated % cells in G1, S, and G2 were then extracted and displayed using Graphpad Prism software, v9.1.0.

### Flow cytometry

After drug treatment, all adherent cells were detached with trypsin-EDTA, pooled with the non-adeherent cells and washed once with HBSS. BD Pharmingen FITC Annexin V Apoptosis Detection Kit II (#556570) was used to stain the cells, and per manufacturer’s instructions. BD Accuri C6 was used for flow signal detection and analysis. Raw data were first gated to exclude debris and doublets by using forward scatter and side scatter gating. Annexin V-positive versus negative and PI-positive versus negative gates were set by observing the untreated plots and identifying the positive and negative signal populations (see supplemental raw data). Identical gating was used on the treated specimens. Percent cells in each quadrant was quantified. The combined Annexin V-positive cells were counted as apoptotic. Data were transcribed to Graphpad Prism software, v9.1.0.

### Xenograft tumor experiments

All animal experiments were approved by the University of Pittsburgh Institutional Animal Care and Use Committee. Female 5- to 6-week-old athymic nude mice (Nu/NJ, Charles River) were housed in a sterile environment with micro isolator cages and allowed access to water and chow *ad libitum*.

Cell line xenografts were established by subcutaneously injecting 5 million WT, *DR5*-KO, or *PUMA*-KO HCT116 cells into both flanks of nude mice. Tumors were allowed to grow for 7 days before treatment. Tumor-bearing mice were randomized into 2 groups and subjected to the following treatments: (i) untreated; (ii) NEO2734 (o.g.; 30 mg/kg on days 1, 5, and 9). NEO2734 was dissolved in 40% PEG400 (Sigma) in distilled water. Tumors were taken for western blotting and histology after 9 days of treatment. Tumors for monitoring growth were followed for up to 3 weeks. Tumor growth was monitored by calipers, and tumor volumes were calculated according to the formula: volume = ½ × length × width × width. Ethical endpoint was defined as a time point when a tumor reached 1.5 cm or more in any dimension. Tumors and vital organs were dissected, fixed in 10% neutral buffered formalin overnight, and embedded in paraffin. Terminal deoxynucleotidyl transferase mediated dUTP Nick End Labeling (TUNEL; ApopTag S7110; EMD Millipore), active caspase-3 (Cell Signaling Technology), active caspase-8 (Cell Signaling Technology), and Ki67 (Dako) immunostaining was performed on 5 μm paraffin-embedded tumor sections as described previously (31), by using an AlexaFluor 488–conjugated secondary antibody (Invitrogen) for detection and 4′6-Diamidino-2-phenylindole (DAPI) for nuclear counter staining. H&E staining was performed using Hematoxylin 7211 and Eosin-Y (Richard Allen Scientific).

### Statistical analysis

Statistical analysis was carried out using GraphPad Prism, v9.1.0. For cell culture experiments, *P* values were calculated using the Student’s *t* tests. Means ± SD are reported in the figures. For animal experiments, *P* values were calculated by repeated measures (RM) ANOVA with Fisher LSD *post hoc* tests. Sample size of 6 mice per group was estimated based on prior experience with active drug treatments on xenograft models. Means ± SEM are reported in the figures. Differences were considered significant if *P* < 0.05.

## Results

### NEO2734 inhibits BRD4 and HAT and potently suppresses CRC cell growth

**Figure 1:**
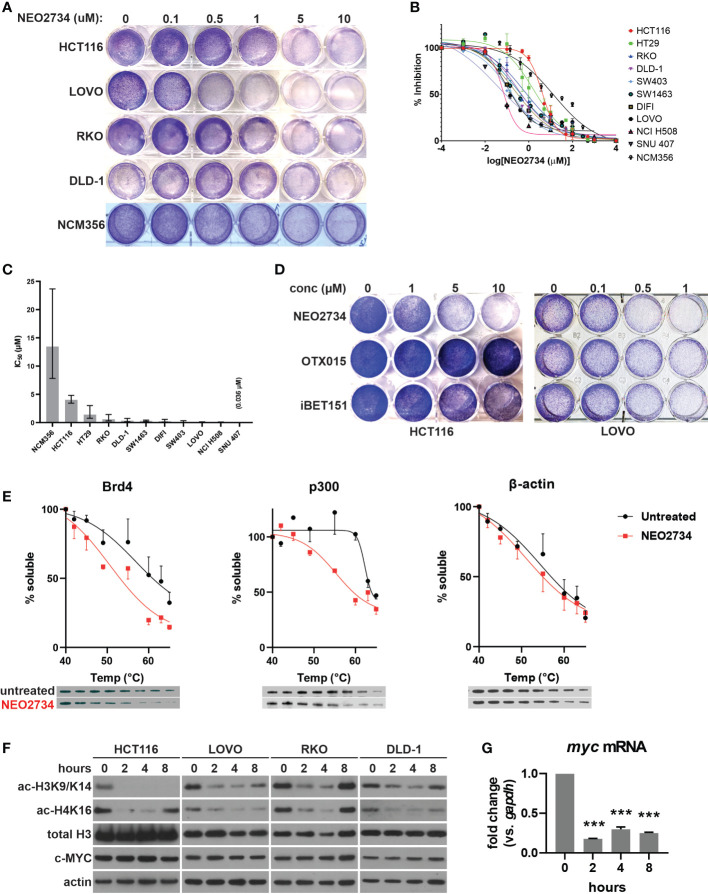
NEO2734 targets histone reader and writer to inhibit histone H3 and H4 acetylation in CRC. **(A)** Crystal violet staining of indicated cell lines were treated with NEO2734 for 72 hours. **(B)** MTS analysis of indicated cell lines treated with NEO2734. **(C)** Average IC_50_ of each cell line. Error bars indicate 95% CI. **(D)** Crystal violet staining of HCT116 and LOVO cells treated with indicated drugs and concentrations for 72 hours. **(E)** Cellular thermal shift assay performed in HCT116 cells. Graphs shows mean soluble protein remaining after incubation at indicated temperatures, with error bars representing +/- SD (only one direction shown for each point for clarity). Representative western blot of each protein and temperature point shown below the graphs. Quantification derived from densitometric measured of 3 independent experiments, with density of each temperature point normalized to the density of a room temp control for that experiment. **(F)** Western blot of acetylated histones (H3K9/K14, H4K16), total histones, and c-MYC in indicated cells over an 8-hour treatment course. **(G)** qRT-PCR of *myc* mRNA in HCT116 cells treated with NEO2734 10µM for the indicated timepoints, normalized to *gapdh* mRNA at each timepoint. ***p < 0.0005 compared to 0h.

We first investigated the therapeutic potential of NEO2734 against CRC cells using ten established CRC cell lines. We found that NEO2734 was able to inhibit growth of all CRC cell lines tested, but was much less active against the normal colon cell line NCM356 (**Figures 1A, B**). The IC_50_ of NEO2734 against the CRC cell lines ranged from nanomolar up to low micromolar concentrations (**Figure 1C**). We observed that the Speckle-type POZ protein (SPOP)-mutant CRC cell lines, NCI-H508 and SNU-407, were particularly sensitive to NEO2734, with IC_50_ of 0.059 µM and 0.036 µM, respectively (**Figures 1B, C**). This finding is consistent with our previous study showing that SPOP-mutated CRCs are sensitized to BET inhibitors (20). We found that NEO2734 was more potent against CRC cells than first generation BET inhibitors alone (**Figure 1D**). Since NEO2734 is a dual inhibitor of BET and HAT proteins, we also investigated its activity against CRC when compared to combined treatment with separate BET and HAT inhibitor drugs. Interestingly, we found that single agent NEO2734 more potently suppressed CRC cells than combined treatment with BET and HAT inhibitor drugs (**Figure S1A**). These findings demonstrate that NEO2734 is preferentially effective against CRC cells compared to normal colonic epithelial cells, and that NEO2734 is more potent than first generation BET inhibitors against CRC cells.

NEO2734 was designed to target both the BET protein BRD4, as well as the HAT protein, also known as p300/CBP (19). We began our investigation of the mechanisms of action by examining whether both protein targets were affected by NEO2734 in CRC cells. Using a cellular thermal shift assay, we observed a decrease in the stability of endogenous BET and HAT proteins in CRC cells that were treated with NEO2734 (**Figure 1E**). This finding suggests that NEO2734 indeed interacts with both BET and HAT proteins in CRC cells. We further examined the proximal downstream targets of inhibition of histone acetylation. We observed decreased acetylation of histones H3K9/K14 and H4K16 occurring within 2 hours of treatment with NEO2734, suggesting that HAT catalytic activity is rapidly suppressed by NEO2734 (**Figure 1F**). Of note, only modest changes in the amount of BET and HAT proteins were seen in the short term (**Figure S1B**), suggesting the rapid changes in histone acetylation are indeed due to decreased HAT activity rather than loss of HAT expression. Interestingly, we observed decreased histone acetylation in several CRC cell lines with different driver mutation profiles, including cells that had mutant p53, KRAS, BRAF, and PIK3CA (32). In contrast to the histone acetylation markers, we observed very little decrease in c-MYC protein expression during the course of treatment with NEO2734, in both shorter-term and longer-term treatment (**Figures 1F**, **S1C**). However, we detected a significant decrease in MYC mRNA which occurred as soon as 2 hours of treatment and was maintained through 8 hours of treatment (**Figure 1G**). These results suggest that NEO2734 engages its intended targets of BRD4 and p300, which in turn inhibits histone acetylation, while causing a decrease of *myc* expression. Further, our results suggest that well-established driver mutations in CRC cells do not predict the sensitivity of the cells to NEO2734.

### NEO2734 induces apoptosis in CRC cells

**Figure 2:**
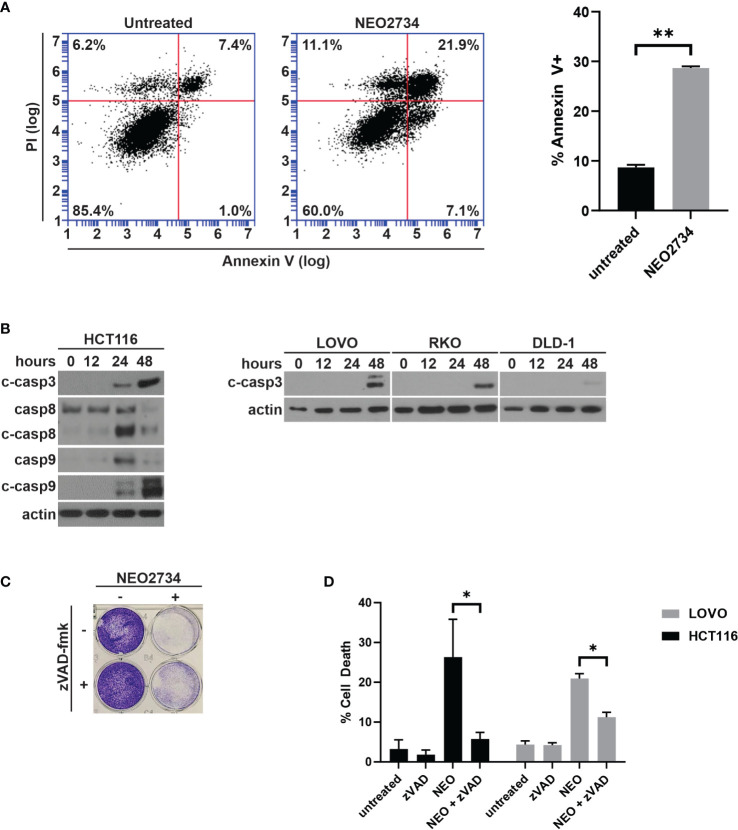
NEO2734 induces apoptosis in CRC cells. **(A)** Annexin V flow cytometry of HCT116 cells after 24 h of treatment with NEO2734 10µM or DMSO control. **(B)** Western blot of cleaved caspase 3, total and cleaved caspase 8, and total and cleaved caspase 9, in HCT116 cells treated with NEO2734 10µM for indicated times. Cleaved caspase 3 also shown for LOVO, RKO, and DLD-1 cells. **(C)** Crystal violet staining of HCT116 cells treated with NEO2734 10µM or control, with or without zVAD-fmk 10µM. Cells treated with zVAD-fmk and NEO2734 were pre-treated with zVAD for 1 hour prior to adding NEO2734. **(D)** Apoptosis percentage of HCT116 cells treated with NEO2734 10µM or control DMSO, with or without zVAD-fmk 10µM co-treatment, as measured by percent fragmented nuclei. All bar plots represent mean of 3 experiments +/- SD (*p < 0.05; **p < 0.005).

Despite limited effect of NEO2734 treatment on c-MYC protein expression, we observed a large number of detached cells during treatment with NEO2734, suggesting that apoptosis may be a mechanism of NEO2734 cytotoxicity. We investigated NEO2734-induced apoptosis further by measuring the fraction of Annexin V positive cells after treatment, and found that NEO2734 caused a significant increase in both of these markers of apoptosis (**Figure 2A**). Consistent with this finding, we observed induction of cleaved caspase 3, caspase 8, and caspase 9 in NEO2734-treated CRC cells (**Figure 2B**). Co-treatment with the pan-caspase inhibitor, zVAD-fmk, was able to partially rescue CRC cells from apoptosis and cytotoxicity caused by NEO2734 (**Figures 2C, D**), suggesting that caspase-dependent apoptosis partially contributes to NEO2734 cytotoxicity. Of note, flow cytometry revealed only a modest change in the cell cycle profile of CRC cells treated with NEO2734 (**Figure S2**), suggesting that inhibition of proliferation is not a major therapeutic mechanism. These results suggest that NEO2734 induces caspase dependent apoptosis as a therapeutic mechanism.

### NEO2734 induces p53 and PUMA-dependent apoptosis in CRC cells

**Figure 3:**
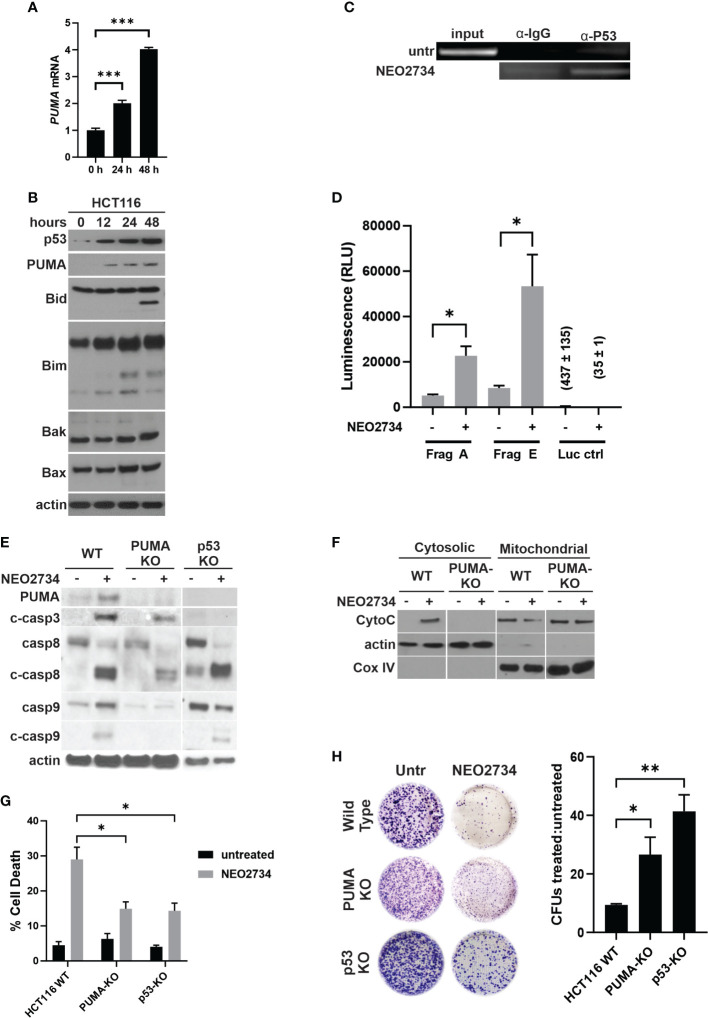
NEO2734 induces apoptosis through p53/PUMA. **(A)** RT-PCR of *PUMA* in HCT116 cells treated with NEO2734 10µM or DMSO for 24 h, normalized to GAPDH. **(B)** Western blot of p53 and PUMA in HCT116 cells treated for up to 48 hours with NEO2734 10µM or DMSO. **(C)** ChIP analysis of p53 binding to the *PUMA* promoter in HCT116 cells treated with NEO2734 10uM for 24 h, with rabbit IgG control IP. *PUMA* promoter was detected by PCR amplification of precipitated DNA fragments using primers flanking the p53 binding site, followed by visualization on agarose gel electrophoresis. **(D)** Luciferase reporter assay of the p53/*PUMA* promoter. HCT116 cells were transfected with pBV-based *PUMA* firefly luciferase reporter plasmids as previously published (20), along with CMV-βgal plasmid. 24 hours after transfection, cells were treated with NEO2734 10µM or DMSO. 24 hours after drug treatment, cell lysates were prepared and luminescence was measured. Signals were normalized for cell viability as well as transfection efficiency based on co-transfected βgal. **(E)** Western blot of PUMA and caspases in parental HCT116 (WT), HCT116 *PUMA*-KO, and HCT116 *p53*-KO cells treated with NEO2734 10µM for 48 hours. **(F)** Western blot of cytosolic versus mitochondrial fractions of HCT116 WT and *PUMA*-KO cells treated with NEO2734 10µM for 48 hours, with antibodies against cytochrome C (CytoC) and cytochrome oxygenase IV (Cox IV). **(G)** Apoptosis percentage as measured by quantification of fragmented nuclei in HCT116 parental and knockout cells treated with NEO2734 10µM for 48 hours. **(H)** Crystal violet staining of colony formation assay of HCT116 parental and knockout cells treated with NEO2734 10µM for 24 h and then replated at 1:400 dilution. Bar graph represents ratio of colonies from NEO2734 treated cultures versus DMSO cultures treated in parallel, for 24 hours. Mean and S.D of 3 independent experiments are shown (*p < 0.05; **p < 0.005; ***p < 0.0005).

NEO2734 induced both caspase 8 cleavage and caspase 9 cleavage, suggesting that both the intrinsic and extrinsic pathways of apoptosis were activated. Intrinsic apoptosis is frequently mediated by the genotoxic stress sensor, p53, as well as its proximal downstream target, p53-upregulated mediator of apoptosis (PUMA) (33). We observed induction of both p53 and PUMA at the mRNA and protein levels by NEO2734 (**Figures 3A, B**, **S3A**). Increased p53 activity has been shown to upregulate pro-apoptosis proteins and downregulate anti-apoptosis proteins in a coordinated fashion (29). We observed increased expression of the pro-apoptosis marker PUMA, along with decreased expression of the anti-apoptosis markers Bcl-2 and Bcl-xL (**Figures 3B**, **S3**). In addition, we observed other markers of apoptosis, including BID cleavage, increased BIM expression, and increased BAK/BAX expression (**Figure 3B**). PUMA has been shown to be a key downstream mediator of p53-dependent apoptosis. We found that NEO2734 treatment induces p53-mediated transcriptional activation of PUMA, as demonstrated by increased binding of p53 protein to the PUMA promoter with chromatin immunoprecipitation, and increased expression of a PUMA reporter assay (**Figures 3C, D**). To investigate if induction of p53/PUMA is necessary for NEO-induced apoptosis, we used *p53* and *PUMA* knockout (KO) cell lines. As expected, the KO cells demonstrated strongly decreased PUMA expression as compared to parental cells (**Figure 3E**). We also observed a decreased ability by NEO2734 to upregulate PUMA expression in both KO cell lines, as expected (**Figure 3E**). Furthermore, we detected decreased cleavage of caspases 3, 8, and 9 after NEO2734 treatment in the KO cells as compared to parental cells, consistent with the attenuation of apoptosis induction (**Figure 3E**). We also observed that cytochrome C was released from the mitochondria into the cytoplasm upon treatment with NEO2734 (**Figure 3F**), consistent with its role in intrinsic apoptosis. NEO2734-induced cytochrome C release was greatly diminished in *PUMA*-KO cells as compared to wild type, further suggesting that intrinsic apoptosis is a key mechanism of NEO2734 cytotoxicity. Both the *p53*-KO and *PUMA*-KO cells demonstrated attenuation of apoptosis induction after NEO2734 treatment, as compared to the parental CRC cells (**Figure 3G**). Furthermore, we found that the relative colony forming potential of the KO cells after NEO2734 treatment was greater than that of the parental cells after treatment (**Figure 3H**). These results suggest that NEO2734 therapeutic activity is partially dependent on p53- and PUMA-mediated intrinsic apoptosis.

### NEO2734 induces DR5-dependent apoptosis in CRC

**Figure 4:**
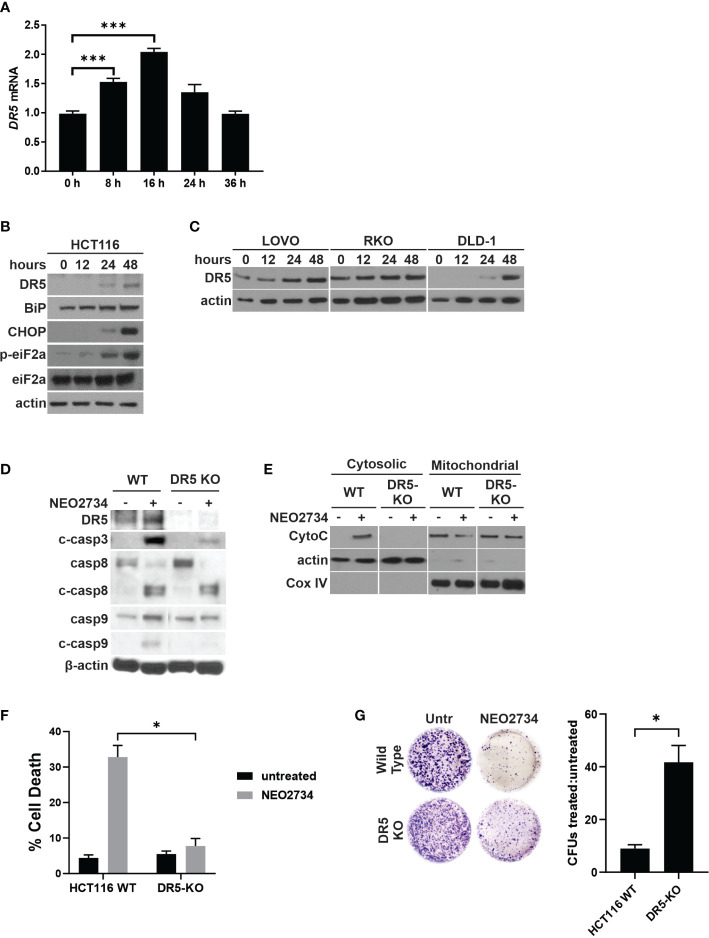
NEO2734 induces DR5-dependent apoptosis. **(A)** RT-PCR of *DR5* in HCT116 cells treated with NEO2734 10µM for the indicated times, normalized to GAPDH. **(B)** Western blotting of DR5 and indicated ER stress response markers in HCT116 cells treated with NEO2734 10µM. **(C)** Western blotting of indicated cell lines for DR5 after treatment with NEO2734. LOVO cells were treated with NEO2734 1µM, RKO and DLD-1 were treated with NEO2734 10µM. **(D)** Western blot of DR5 and caspases in parental HCT116 (WT) and HCT116 *DR5*-KO cells treated with NEO2734 10µM for 48 hours. **(E)** Western blot of cytosolic versus mitochondrial fractions of HCT116 WT and *DR5*-KO cells treated with NEO2734 10µM for 48 hours, with antibodies against cytochrome C (CytoC) and cytochrome oxygenase IV (Cox IV). **(F)** Apoptosis percentage as measured by quantification of fragmented nuclei in HCT116 parental and knockout cells treated with NEO2734 10µM for 48 hours. **(G)** Crystal violet staining of colony formation assay of HCT116 parental and knockout cells treated with NEO2734 10µM for 24 h and then replated at 1:400 dilution. Bar graph represents ratio of colonies from NEO2734-treated cultures versus DMSO cultures treated in parallel, for 24 hours. Mean and S.D of 3 independent experiments are shown (*p<0.05; ***p<0.0005).

Due to NEO2734 suppression of CRC cells being only partially dependent on intrinsic apoptosis, as well as the observation that caspase 8 cleavage is induced by NEO2734, we hypothesized that NEO2734 also induces extrinsic apoptosis through the death receptor pathway. We have previously shown that BET inhibitors upregulate the extrinsic pathway by activating transcription of *Death Receptor 5* (*DR5*), and that CRC cell suppression by the BET inhibitor JQ1 is mostly dependent on DR5-mediated apoptosis (20). In the present study, we found that NEO2734 induces DR5 at the RNA and protein levels (**Figures 4A–C**). We also observed induction of ER stress markers BiP, p-eiF2a, and CHOP (**Figure 4B**). ER stress activation has been shown to activate death receptors (34). To investigate if DR5 activation is required for NEO2734-induced apoptosis, we used a *DR5*-KO cell line (35). We observed decreased activation of caspases 3, 8, and 9 in *DR5*-KO cells treated with NEO2734 (**Figure 4D**). Loss of DR5 also strongly inhibited the release of cytochrome C in response to NEO2734 treatment (**Figure 4E**). Similar to the *p53*- and *PUMA*-KO cells, *DR5*-KO cells demonstrated an attenuated apoptosis response to NEO2734 (**Figure 4F**). Finally, knockout of DR5 was also able to partially rescue the colony forming potential of NEO2734-treated CRC cells (**Figure 4G**). These results suggest that in addition to p53/PUMA, DR5 signaling is also partially required for NEO2734-induced apoptosis.

### NEO2734 cytotoxicity against CRC cells depends on both p53/PUMA and DR5-mediated apoptosis

**Figure 5:**
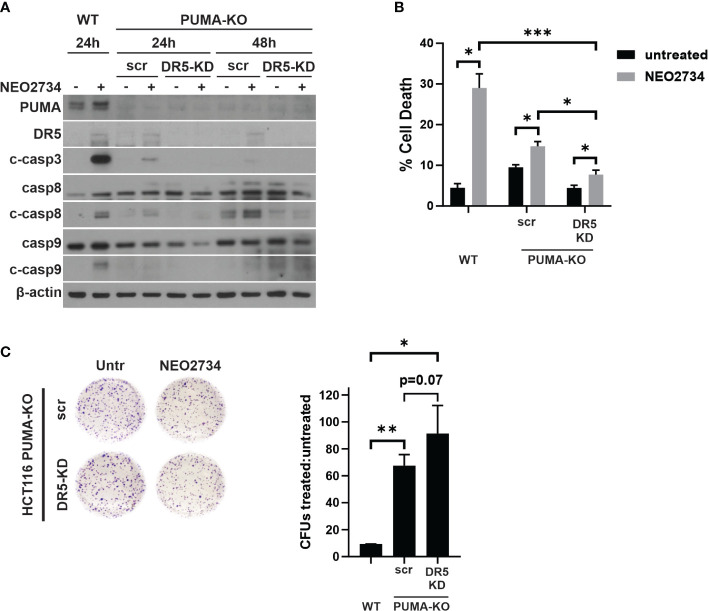
NEO2734 activity depends on both PUMA- and DR5- mediated apoptosis. **(A)** Western blot of indicated HCT116 cell lines after transfection with either scrambled (scr) or DR5 targeted (DR5-KD) siRNA, followed by treatment with NEO2734 10µM. Cells were transfected 24 hours after plating and then treated with drug 24 hours after transfection. Times are the hours of drug treatment. **(B)** Apoptosis percentage as measured by quantification of fragmented nuclei in HCT116 parental (WT) and PUMA-KO cells. PUMA-KO cells were transfected with either control (scr) or DR5-targeted siRNA (DR5-KD) at 24 hours after plating, and then treated with drug 24 hours after transfection. All cells were treated with NEO2734 10µM for 48 hours. **(C)** Crystal violet staining of colony formation assay of HCT116 parental and PUMA-KO cells treated with NEO2734 10µM for 24 h and then replated at 1:400 dilution. Bar graph represents ratio of colonies from NEO2734-treated cultures versus DMSO cultures treated in parallel, for 24 hours. Mean and S.D of 3 independent experiments are shown (*p < 0.05; **p < 0.005; ***p < 0.0005).

We found that loss of p53/PUMA or loss of DR5 was able to partially block NEO2734-induced apoptosis. Thus, we hypothesized that both pathways are activated by NEO2734 treatment to induce apoptosis. To test if dual blockade of these pathways was able to additively attenuate NEO2734-induced apoptosis, we investigated NEO2734 treatment of *PUMA*-KO cells transfected with a *DR5* siRNA (KO/KD cells). We first confirmed that the KO/KD cells were indeed depleted of PUMA and DR5 by western blotting (**Figure 5A**). Importantly, these KO/KD cells demonstrated an even greater suppression of caspase cleavage than PUMA-KO cells, after NEO2734 treatment (**Figure 5A**). We also found that concurrent loss of DR5 and PUMA caused a greater decrease in NEO2734-induced apoptosis (**Figure 5B**). Furthermore, we found that the KO/KD cells had further increased colony formation potential as compared to the PUMA-KO cells (**Figure 5C**). Together, these results suggest that the intrinsic and extrinsic apoptosis pathways work in parallel during induce NEO2734-induced apoptosis.

### NEO2734 induces CRC apoptosis *in vivo*

**Figure 6:**
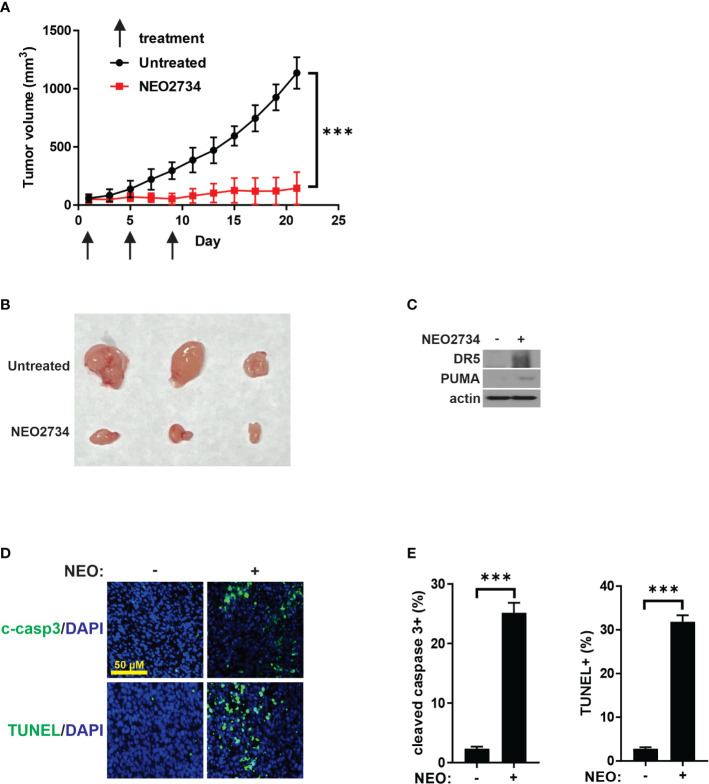
NEO2734 induces CRC apoptosis *in vivo*. **(A)** Growth curves of HCT116 tumor xenografts implanted in flanks of nude mice. Treatments were started after tumors reached 50-100 mm^3^ in size. Tumor volumes were estimated by the formula V=w^2*l/2 (V=volume, w=width or short dimension of tumor, l=length or long dimension of tumor). Results are the mean and S.D. of 6 independent tumors. **(B)** Representative photographs of NEO2734-treated and control HCT116 xenograft tumors dissected from mice after sacrifice on day 21. **(C)** Western blot of HCT116 xenograft tumors with indicated antibodies. **(D)** Representative immunofluorescence staining of indicated markers in control versus NEO2734-treated xenografts. **(E)** Quantification of c-casp3 and TUNEL positive cells in xenografts. Mean and S.D. of 6 independent tumors are shown (***p < 0.0005).

To investigate the *in vivo* activity of NEO2734 against CRC, we treated nude mice bearing HCT116 xenografts. Based on published mouse experiments (22) and our own experience with NEO2734 single dose safety (data not shown), we initially treated mice with 5 consecutive days of NEO2734 at 30 mg/kg by oral gavage (OG). These mice demonstrated significant hematologic and gut toxicity (**Figure S4A**) along with weight loss (data not shown). Therefore, we established NEO2734 30 mg/kg, 3 doses given once 4 days as the optimal dose for treatment of mice (**Figure S4B**). HCT116 tumors in mice treated with NEO2734 at this safer regimen demonstrated significantly slower tumor growth as compared to tumors in control mice (**Figures 6A, B**). We observed increased DR5 and PUMA expression in these tumors, consistent with our *in vitro* findings (**Figure 6C**). We also saw significantly increased tumor apoptosis after NEO2734 treatment *in vivo* (**Figures 6D, E**). These findings indicate that NEO2734 potently suppresses CRC cells and induces both p53/PUMA- and DR5-dependent apoptosis *in vivo*.

## Discussion

BET inhibitors are a novel class of epigenetic agents with promising activity in several liquid and solid tumors. The most proximal targets of these drugs are bromodomain proteins or histone “readers”, primarily the oncogenic BRD4 protein. We sought to understand the activity of the BET inhibitor NEO2734 in the treatment of CRC. NEO2734 generally appears to be more potent against CRC than earlier generation BET inhibitors such as iBET-762 and OTX-015, although in some CRC models we studied, this improvement was modest. NEO2734 is also an inhibitor of HATs or histone “writers”. Short term treatment with NEO2734 does appear to inhibit HAT activity in CRC. The antibodies we used for detecting acetylated histone markers have been shown to be more specific for detecting acetylation at histones H3K9, H3K14, and H4K16. CBP and p300 have been previously shown to acetylate H3K14 and H4K16 in biochemical assays (36). However, we also detected an increase in acetylation levels following an initial transient decrease. Although our study does not reveal the cause for this transient suppression of histone acetylation, it could be due to a homeostatic mechanism that restores promoter histone acetylation following small molecule inhibition of CBP/p300 (37). While we did not find a significant suppression of c-MYC protein expression after NEO2734 treatment, we did observe a rapid and significant decrease in *myc* mRNA expression with treatment. c-MYC frequently is subject to many regulatory mechanisms and suppression of just the BET/HAT pathway may not be sufficient to eliminate c-MYC expression. Further, the decrease in MYC mRNA appears to be insufficient to significantly reduce protein expression, suggesting that c-MYC protein translation or stability may be able to compensate for this decrease in mRNA expression. Importantly, we show that the dual inhibitor NEO2734 is consistently a more potent suppressor of CRC cells than first generation BET inhibitors.

Our study led us to characterize one of the mechanisms of cytotoxicity of NEO2734 in CRC, which appears to be apoptosis *via* both the intrinsic and extrinsic pathways. Many chemotherapies and targeted therapies eventually cause some form of regulated cell death as a terminal mechanism of cytotoxicity (38). In this case, NEO2734 appears to exert its cytotoxic effect through two distinct yet interconnected apoptosis pathways. Interestingly, the blockade of either of these pathways alone was insufficient to completely suppress the apoptosis caused by NEO2734, suggesting that these two apoptosis pathways could act in parallel to ensure killing of tumors. This property of NEO2734 may give it a therapeutic advantage over other BET inhibitors in the treatment of CRC. Interestingly, we demonstrated that a key step of intrinsic mitochondrial apoptosis, the release of cytochrome C from the mitochondria to the cytoplasm, was impaired in the DR5-KO cells. These findings suggest that there is significant crosstalk between the intrinsic and extrinsic branches of apoptosis during NEO2734 treatment. Concurrent inhibition of both of these pathways still did not completely suppress NEO2734-induced cell death. Thus, further studies on alternate mechanisms of regulated cell death, such as necroptosis or ferroptosis, are warranted.

An important factor that emerged in our investigation was the narrow therapeutic window of NEO2734 against CRC. We were able to identify a safe regimen for the treatment of xenograft-bearing mice, but our initial regimen of daily NEO2734 treatments proved to be too toxic to use. This experience parallels the ongoing clinical experience with first generation BET inhibitors, which in clinical trials have demonstrated significant treatment-related adverse events (39–43). We suspect that toxicities and narrow therapeutic windows may be class-wide properties of BET inhibitors. Consistent with phase 1 clinical trials of BET inhibitors in humans, we observed a narrow therapeutic window due to GI and hematologic toxicity. Further investigation into methods for predicting BET inhibitor sensitivity and mitigating toxicity are warranted.

Biomarkers that can be used to predict response or resistance to specific treatments have become an important part of clinical oncology. mCRC is a biologically heterogeneous disease, and recently approved treatment options have all hinged on our understanding of a well-characterized molecular subtype of CRC with clinical biomarkers. The approvals of pembrolizumab for MSI-H mCRC (4, 44) and cetuximab plus encorafenib for BRAF V600E mCRC (45, 46) are a few high-profile examples of such biomarker-driven therapeutic breakthroughs in mCRC. One interesting biomarker that has emerged for BET inhibitors is mutation of SPOP, a component of the E3 ubiquitin ligase complex that is instrumental in regulating BRD4 stability. In prostate and endometrial cancers, SPOP mutations appear to confer either sensitivity or resistance against first generation BET inhibitors, depending on the tumor type (22, 47, 48). In CRC, our group previously showed that SPOP mutations, present in ~1% of mCRC, may confer sensitivity to first generation BET inhibitors (20). In this study, we demonstrate that SPOP mutant CRC cells are significantly more sensitive to NEO2734 than first generation BET inhibitors. Thus, SPOP mutation is a promising biomarker for BET inhibitor sensitivity that warrants further investigation.

## Data availability statement

The raw data supporting the conclusions of this article will be made available by the authors, without undue reservation.

## Ethics statement

The animal study was reviewed and approved by UPMC IACUC.

## Author contributions

CK: Concept, experimental design, experimental conduct, manuscript drafting, manuscript revision. JT: Experimental design, experimental conduct. KE: Experimental conduct, manuscript revision. MC, FD, and SH: Experimental conduct. FG: Concept, manuscript revision. YH: Experimental design, manuscript revision. JY: Funding support, manuscript revision. LZ: Concept, experimental design, funding support, manuscript revision. All authors contributed to the article and approved the submitted version.

## Funding

This work was supported by the U.S. National Institutes of Health grants (T32CA193205 to CK, R01CA247231 and R01CA248112 to LZ; R01CA215481 to JY; R01CA260357 to YH). This project used the UPMC Hillman Cancer Center Animal Facility, Cytometry Facility, and Tissue and Research Pathology Services, which are supported in part by P30CA047904.

## Acknowledgments

NEO2734 was provided by NEOMED Therapeutics 1, Inc.

## Conflict of interest

FG is a consultant to Epigene Therapeutics.

The remaining authors declare that the research was conducted in the absence of any commercial or financial relationships that could be construed as a potential conflict of interest.

## Publisher’s note

All claims expressed in this article are solely those of the authors and do not necessarily represent those of their affiliated organizations, or those of the publisher, the editors and the reviewers. Any product that may be evaluated in this article, or claim that may be made by its manufacturer, is not guaranteed or endorsed by the publisher.
