# Supplementary material for: Dual inhibition of BET and HAT/p300 suppresses colorectal cancer via DR5- and p53/PUMA-mediated cell death
Source: Front Oncol. 2022 Oct 12;12:1018775. doi: 10.3389/fonc.2022.1018775 (PMC9599411; doi:10.3389/fonc.2022.1018775)
Supplement: Supplementary file 1 [file DataSheet_1.docx]

**Supplemental figure legends**

Figure S1. A) MTS analysis of indicated cell lines treated with NEO2734, the first generation BET inhibitor OTX-015, the histone acetyltransferase inhibitor CPI-637, or combination OTX-015 and CPI-637. B) Western blot of p300 and BRD4 in HCT116 cells treated with NEO2734 for the indicated times. C) Western blot of acetylated histones H3 and H4, total H3, and c-MYC in indicated cell lines treated with NEO2734.

Figure S2. A) Cell cycle distribution of HCT116 cells treated with NEO2734 10µM for 24 hours. B) Cell cycle profile based on propidium iodide flow cytometry of HCT116 cells treated with NEO2734 for 10µM for 24 hours. Watson model from Flowjo v10 was used to estimate fractions of cells in each phase of cell cycle. See supplemental raw data for details on modeling parameters.

Figure S3. A) Western blot of p53 and PUMA in indicated cell lines after treatment with NEO2734 10µM for indicated times. B) Western blot of Bcl-2, Bcl-xL, Mcl-1, and Noxa in HCT116 cells treated with NEO2734 10µM for the indicated times.

Figure S4. A) Representative H&E-stained sections of spleen, small bowel, and colon from mice treated with vehicle or daily NEO2734 (30 mg/kg daily, OG). Tissues were obtained after 5 consecutive days of treatment. B) Average weight of mice treated with the final established dose of NEO2734 (30 mg/kg, on days 1, 5, and 9) over the course of the experiment (N=6).

**Supplemental materials and methods**

Antibodies:

| **Target** | **Manufacturer** | **Catalog No.** | **Dilution** |
| --- | --- | --- | --- |
| PUMA | Custom^1^ | See ref 1 | 1:1000 |
| DR5 | ProSci | 2019 | 1:2000 |
| β-Actin | Sigma | A5441 | 1:4000 |
| P53 | Santa Cruz | Sc-126 | 1:1000 |
| ac-H3 (K9/K14) | Millipore | 06-599 | 1:2000 |
| ac-H4 (K16) | Millipore | 07-329 | 1:1000 |
| H3 | Abcam | Ab1751 | 1:1000 |
| c-MYC | Abcam | Ab39688 | 1:1000 |
| c-CASP3 | Cell Signaling | 9661 | 1:1000 (WB), 1:100 (IF) |
| c-CASP8 | Cell Signaling | 9746 | 1:1000 |
| c-CASP9 | Cell Signaling | 9502 | 1:1000 |
| BIP | Cell Signaling | 3177 | 1:1000 |
| ATF4 | Santa Cruz | Sc-200 | 1:1000 |
| CHOP | Cell Signaling | 2895 | 1:1000 |
| p-EIF2α | Cell Signaling | 3398 | 1:500 |
| EIF2α | Santa Cruz | Sc-11386 | 1:1000 |
| BCL-2 | Millipore | OP60 | 1:1000 |
| BCL-XL | Novus Biologicals | NBP1-28559 | 1:1000 |
| MCL-1 | Cell Signaling | 39224 | 1:1000 |
| NOXA | Oncogene Science | OP180 | 1:2000 |
| BID | Cell Signaling | 2002 | 1:1000 |
| BAX | Santa Cruz | Sc-493 | 1:1000 |
| BAK | Upstate | 06-536 | 1:1000 |
| BAD | Cell Signaling | 9292 | 1:1000 |
| BRD4 | Abcam | Ab128874 | 1:1000 |
| P300 | Santa Cruz | Sc-584 | 1:1000 |

Primer sequences:

| **Experiment** | **Gene/orientation** | **Sequence** |
| --- | --- | --- |
| qRT-PCR | *gapdh*/forward | 5’-GTCTCCTCTGACTTCAACAGCG-3’ |
| qRT-PCR | *gapdh*/reverse | 5’ACCACCCTGTTGCTGTAGCCAA-3’ |
| qRT-PCR | *bbc3*/forward | 5’-CGACCTCAACGCACAGTACGA-3’ |
| qRT-PCR | *bbc3*/reverse | 5’-AGGCACCTAATTGGGCTCCAT-3’ |
| qRT-PCR | *dr5*/forward | 5’-AAGACCCTTGTGCTCGTTGT-3’ |
| qRT-PCR | *dr5*/reverse | 5’-AGGTGGACACAATCCCTCTG-3’ |
| qRT-PCR | *myc*/forward | 5’-TCTTGACATTCTCCTCGGTGTCCGAGG-3’ |
| qRT-PCR | *myc*/reverse | R: 5’-TACCCTCTCAACGACAGCAGCTCGCCC-3’ |
| ChIP | *bbc3*/forward | 5’-GATTACAGGCATGCGCCAGA-3’ |
| ChIP | *bbc3*/reverse | 5’-ACCCACACTGATGATCACAC-3’ |

1. Yu, J., Wang, Z., Kinzler, K. W., Vogelstein, B. & Zhang, L. PUMA mediates the apoptotic response to p53 in colorectal cancer cells. *Proc. Natl. Acad. Sci. U. S. A.* **100**, 1931 (2003).
