# Supplementary figures and images for: Dual inhibition of BET and HAT/p300 suppresses colorectal cancer via DR5- and p53/PUMA-mediated cell death
Source: Front Oncol. 2022 Oct 12;12:1018775. doi: 10.3389/fonc.2022.1018775 (PMC9599411; doi:10.3389/fonc.2022.1018775)

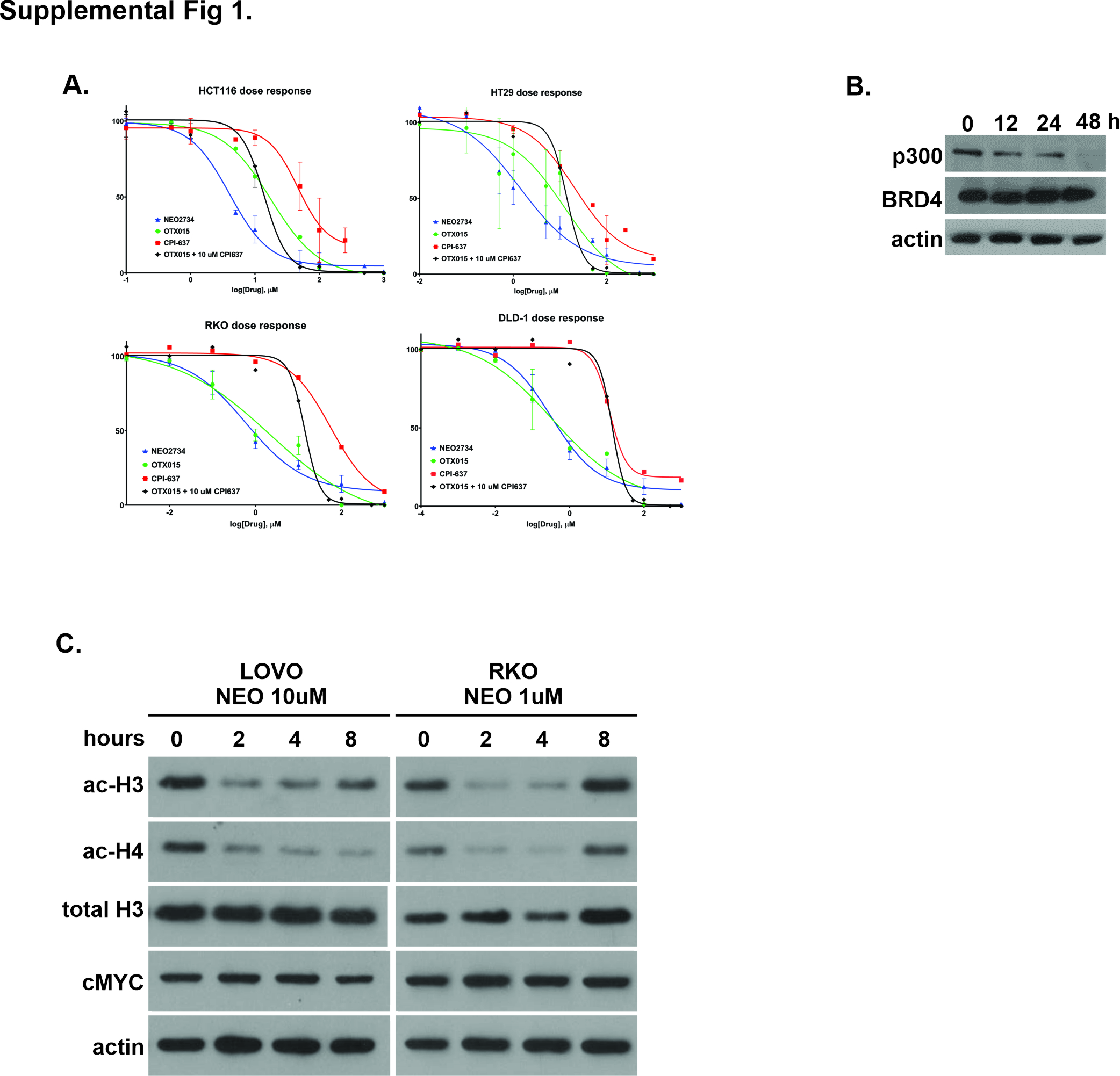

Supplement: Supplementary file 2 [file Image_1.jpg]

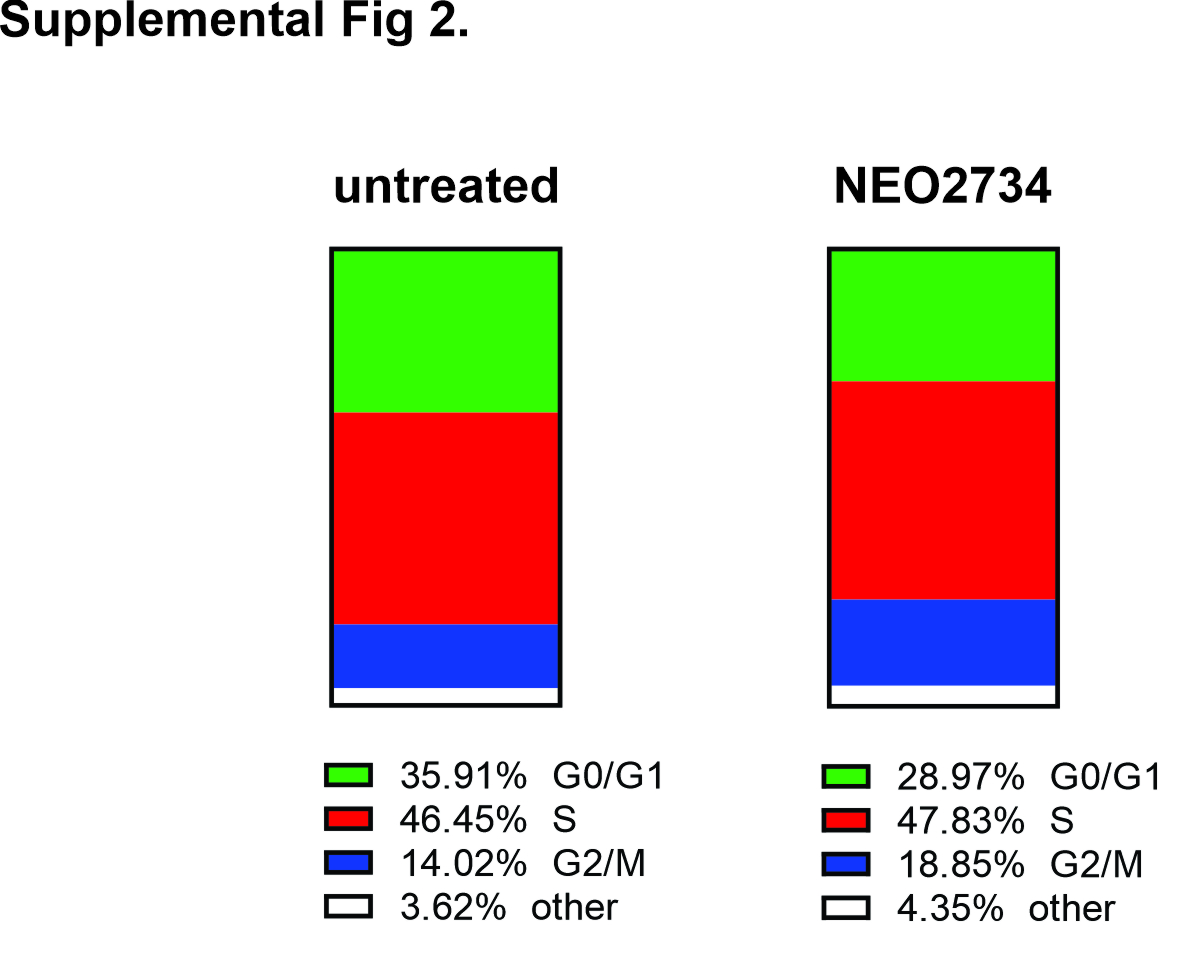

Supplement: Supplementary file 3 [file Image_2.jpg]

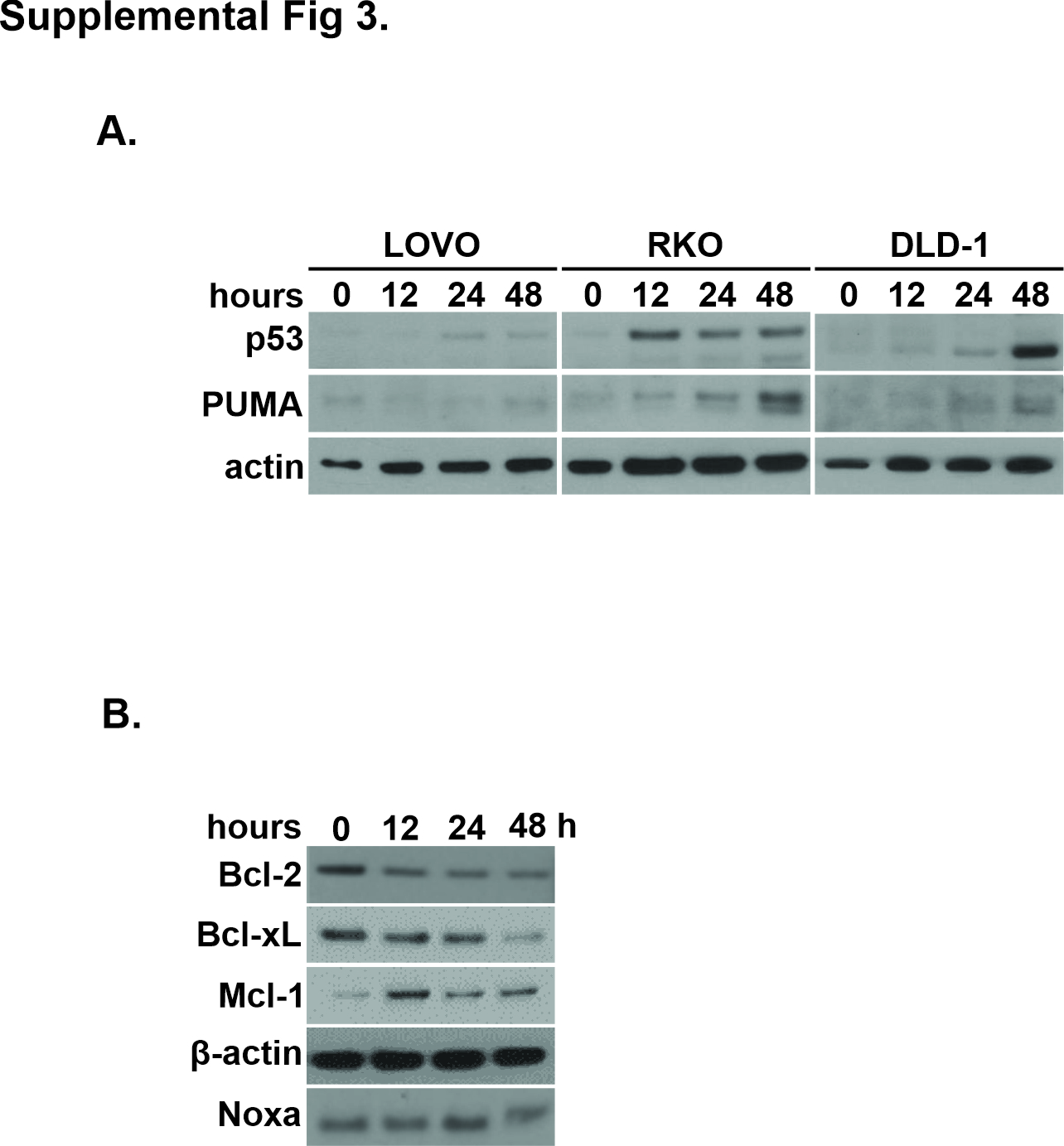

Supplement: Supplementary file 4 [file Image_3.jpg]

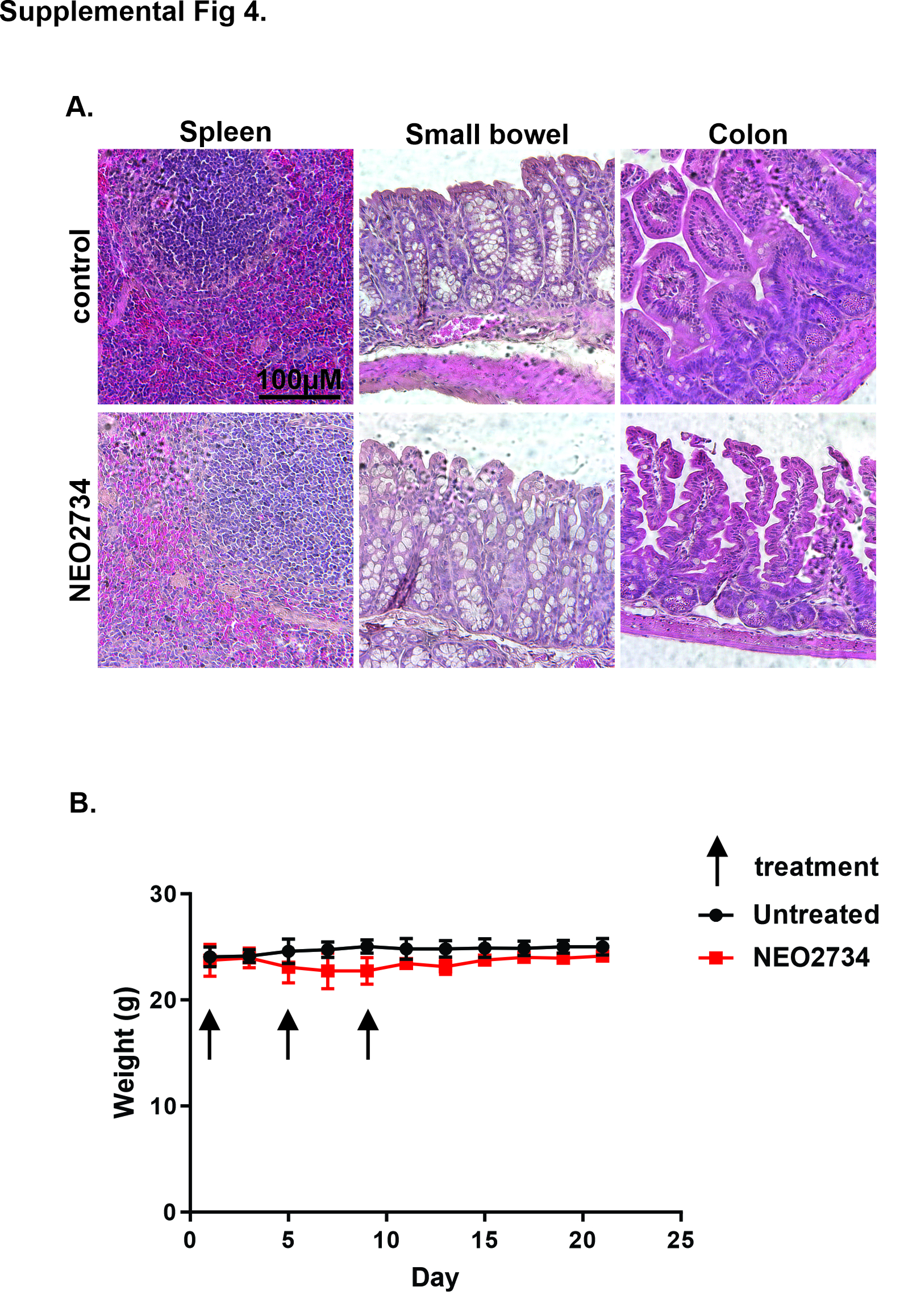

Supplement: Supplementary file 5 [file Image_4.jpg]
